# Increase in Prevalence of Overweight in Dutch Children and Adolescents: A Comparison of Nationwide Growth Studies in 1980, 1997 and 2009

**DOI:** 10.1371/journal.pone.0027608

**Published:** 2011-11-15

**Authors:** Yvonne Schönbeck, Henk Talma, Paula van Dommelen, Boudewijn Bakker, Simone E. Buitendijk, Remy A. HiraSing, Stef van Buuren

**Affiliations:** 1 TNO Child Health, Leiden, The Netherlands; 2 VU University Medical Center Amsterdam, EMGO Institute of Health Care Research, Amsterdam, The Netherlands; 3 TNO Life Style, Leiden, The Netherlands; 4 Leiden University Medical Center, Department of Pediatrics, Leiden, The Netherlands; 5 University of Utrecht, Department of Methodology and Statistics, Utrecht, The Netherlands; UCL Institute of Child Health, University College London, United Kingdom

## Abstract

**Objective:**

To assess the prevalence of overweight and obesity among Dutch children and adolescents, to examine the 30-years trend, and to create new body mass index reference charts.

**Design:**

Nationwide cross-sectional data collection by trained health care professionals.

Participants: 10,129 children of Dutch origin aged 0–21 years.

**Main Outcome Measures:**

Overweight (including obesity) and obesity prevalences for Dutch children, defined by the cut-off values on body mass index references according to the International Obesity Task Force.

**Results:**

In 2009, 12.8% of the Dutch boys and 14.8% of the Dutch girls aged 2–21 years were overweight and 1.8% of the boys and 2.2% of the girls were classified as obese. This is a two to three fold higher prevalence in overweight and four to six fold increase in obesity since 1980. Since 1997, a substantial rise took place, especially in obesity, which increased 1.4 times in girls and doubled in boys. There was no increase in mean BMI SDS in the major cities since 1997.

**Conclusions:**

Overweight and obesity prevalences in 2009 were substantially higher than in 1980 and 1997. However, the overweight prevalence stabilized in the major cities. This might be an indication that the rising trend in overweight in the Netherlands is starting to turn.

## Introduction

Over the past three decades childhood overweight and obesity have reached epidemic proportions in most industrialized countries [1]. In some European countries, such as the Scandinavian countries and the Netherlands, the prevalence of childhood overweight and obesity are lower than in Mediterranean countries. Nonetheless, the proportion of overweight children has also been rising in those countries [2]. In the Netherlands overweight rates almost doubled between 1980 and 1997, from five to nine percent in boys and from seven to twelve percent in girls; obesity rates even tripled from 0.3 to 0.9% and from 0.5 to 1.6% in boys and girls respectively [3], [4]. This increase in prevalence rates will have a substantial economic impact. The WHO estimates that, at present, adult overweight and obesity are responsible for about six percent of health care expenditure in the European Region [5].

Childhood overweight has both psychological and health consequences. In the short run, overweight leads to conditions including high blood pressure, diabetes mellitus type 2, high cholesterol, depressive symptoms and low self-esteem. In the long run, children with overweight or obesity are more likely to become obese as adults [6], [7], which translates into increased risk for chronic diseases, including cardiovascular disease, hypertension, diabetes mellitus type 2 and even premature mortality [8], [9]. In addition, obese adults who were overweight before the age of 8 years are found to be heavier in adulthood than those who became overweight in adolescence or adulthood [10]. A recent study found that body mass index (BMI) change between the ages of two to six years specifically, contributes to adult overweight [11]. Early prevention of childhood overweight is therefore of utmost importance.

Up to date national prevalence rates of overweight and obesity are essential to monitor the development of overweight and obesity. The Netherlands holds a unique position in the world because regular nation-wide growth studies have been conducted since 1955 [12]–[15]. These studies all used similar methodologies for sampling and measuring children up to 20 years of age. The Dutch 1980 study was the largest of the six studies used to calculate the now widely adopted cut-off values for overweight and obesity for children [16].

This paper presents the first results from the Fifth Dutch Growth Study, conducted between 2008–2010, which extends the Dutch tradition in growth studies. This paper reports the current distribution of BMI in Dutch children in the form of BMI reference charts and age-related prevalences for overweight and obesity. We compare these to results obtained in earlier studies. In addition, we study the relationship between BMI and geographical region, educational level of the child and educational level of the parents. Last, we evaluate the trends since the Dutch Growth Study in 1980.

## Methods

### Ethics statement

Data collection of growth studies is part of routine youth health care in the Netherlands [17], and is not regarded as medical research. Before measurement, oral consent was obtained from each child (and parent for children younger than 16 years). Cooperation, or lack thereof, was registered on the questionnaire. Data were analyzed anonymously. The Medical Ethical Review Board of Leiden University Medical Center approved of the study and the way consent was obtained.

### Data sources

The Fifth Dutch Growth Study is a cross-sectional study in which data were collected on the growth of children aged 0–21 years in the Netherlands. The measurements took place between May 2008 and October 2009. The sample was stratified by region (regions of Municipal Health Services (MHS)), sex and age, according to national distributions [18]. Until 4 years of age, measurements were performed in regular periodical health examinations in 28 Well Baby Clinics at the ages of 1, 2, 3, 6, 9, 12, 15, 18, 21, 24, 30, 36 and 45 months. Between the ages of four to eight years, children were measured at 23 MHS offices during two regular preventive health assessments performed at the ages of approximately 5.5 and 7.5 years. From age nine years onward, children received a personal invitation from the MHS after being randomly drawn, stratified for age and sex, from the register of the Municipal Register Office. In addition, we collected measurements within randomly selected primary and secondary schools, in two high schools, two universities and at a youth festival. The collection was supplemented by data from two recent large, high quality studies performed by trained staff at primary schools in Amsterdam (GGD Amsterdam, Amsterdam) and vocational education in the east of the Netherlands (Deltion College Zwolle/OPOZ VU-Windesheim). From these datasets, random samples (n = 270 and n = 342 respectively) were added to the dataset for this study.

The study was approved by the Medical Ethical Review Board of Leiden University Medical Center.

### Exclusion criteria

Exclusion criteria for the Fifth Dutch Growth Study were similar to those for the previous Dutch Growth Studies: children with diagnosed growth disorders and those on medication known to interfere with growth were excluded. In accordance to the growth study in 1997, but in contrast to previous Dutch Growth Studies, infants with a birth weight below 2500 g were included. Children of non-Dutch parents [19] were excluded from the analyses presented in this paper. Results regarding children of Turkish and Moroccan origin will be published elsewhere.

### Measurements

The measurements were standardised and were performed by trained health care professionals. Infants' length was measured to the nearest 0.1 cm in the supine position until two years of age. From two years of age, standing height was measured to the nearest 0.1 cm. Infants up to 15 months of age were weighed naked on calibrated baby scales. Older children were weighed wearing underwear only, on calibrated mechanical or electronic step scales. Weight was rounded to the nearest 0.01 kg for infants and to the nearest 0.1 kg for older children. A questionnaire, filled in by a health care professional, was used to collect demographic variables.

### Variable definitions

The sample was clustered into four geographical regions: North (Friesland, Groningen, Drenthe), East (Overijssel, Gelderland, Flevoland), West (Noord-Holland, Zuid-Holland, Utrecht – not including the major cities) and South (Zeeland, Noord-Brabant, Limburg). A fifth region was formed by the four largest Dutch cities (Amsterdam, Rotterdam, Utrecht, and The Hague). The educational level of the child was determined at the time of measurement. If an adolescent of over 15 years of age had left the educational system, the highest completed education was recorded. The educational level of the parents was defined as the educational level of the highest educated parent and categorized into low, middle, and high level [20]. BMI was calculated as weight/height^2^ and expressed as kg/m^2^. Overweight and obesity prevalence rates were calculated for 2009, 1997 and 1980 using the International Obesity Task Force (IOTF) cut-off values [16]. All overweight figures in this paper include obesity. For data cleaning purposes and for comparison of BMI changes between 1997 and 2009, standard deviation scores (SDS) per age were calculated using the 1997 references [4], [12], [21], [22].

### Statistical analysis

Data were cleaned using descriptive statistics including frequency tables, contingency tables and scatter plots. Outliers, defined as values over or below 5 SDS were checked for data entry errors and corrected where possible. If no correction was possible, these measurements were considered erroneous and defined as missing. The distributions of educational level of the child and geographical region were compared to the national distributions [23] to check for representativeness. We used the Multivariate Imputation by Chained Equation (MICE) method [24] to correct for educational levels or geographical regions that were underrepresented. The imputation model was based on age, sex, height SDS, weight SDS, waist SDS, hip SDS, parental height, birth weight, ethnicity, socio-economic status score [25], educational level of the child and parents, and geographical region. Passive imputation was performed on the interactions between age and region, age and all SDS, age squared, and age squared and all SDS.

The distribution of BMI in the population depends on age and tends to be positively skewed. Therefore, BMI reference values for 2009 were calculated using the LMS method [26]. This method summarizes the SDS lines by three smooth curves representing skewness (L curve), the median (M curve), and coefficient of variation (S curve). L values of 1 indicate normality and smaller values represent progressively greater skewness. The M curve is the 0 SDS line or 50th centile curve for BMI. The S curve defines the coefficient of variation, and multiplied by 100 it can be interpreted as a percentage. The choice of the smoothing parameters (effective degrees of freedom, edf's) for the L, M, and S curves was made by creating worm plots: local detrended Q-Q plots (“Q” stands for quantile) of the SDS of the reference sample across 16 age groups [27]. The curves were fitted as cubic splines. Finally, the age-related BMI reference values for 2009 were estimated.

The relative proportion of obesity among overweight children was calculated by dividing the proportion of obese children by the proportion of overweight (including obese) children. To determine if the trend in overweight differed between children living inside and outside the major cities, we compared prevalences in both regions between 1997 and 2009 for boys and girls separately and tested for significance by the Chi-square-tests.

To determine if trends in BMI SDS between 1997 and 2009 varied between different geographical regions and between children of low, middle and higher educated parents, we calculated mean BMI SDS (using the 1997 references as described above) with 95% confidence intervals for children aged two years and older for each category of these variables for 1997 and 2009.

R version 2.9.0 with GAMLSS-package [28] was used for the imputation and for estimating the BMI SDS reference values. All other statistical analyses were performed in SPSS version 17.0 for Windows.

## Results

The sample provided 10,030 children of Dutch origin and 1,975 imputed cases, resulting in an analysis sample of 12,005 children (5,811 boys, 6,194 girls) aged 0–21 years. In total, data of 4,382 children aged 0–3 years and of 7,623 children aged 4–21 years were available. Data of 146 children aged 22–25 years were included in the dataset for fitting BMI reference charts, but they were excluded for further analyses.

Table 1 presents the LMS values for BMI by age and sex. It shows that, for example, for boys aged 5 years the median (M) BMI was 15.64 whilst for girls this was 15.62, and that the coefficient of variation (S) of BMI was about 8% in infancy and rose up to 13–14% in adolescence.

**Table 1:** LMS values for BMI (kg/m^2^) in Dutch 0–21 year olds in 2009 by age and sex.

	boys (n = 5,885)			girls (n = 6,219)		
age (y)	L	M	S	L	M	S
0.0767*	1.0597	14.75	0.0858	−0.2714	14.55	0.0839
0.2500	0.7708	16.26	0.0834	−0.2900	15.73	0.0830
0.5000	0.4610	17.06	0.0809	−0.2990	16.63	0.0821
0.7500	0.2431	17.22	0.0792	−0.2968	16.88	0.0814
1.0000	0.0836	17.18	0.0783	−0.3027	16.76	0.0808
1.2500	−0.0462	17.05	0.0778	−0.3309	16.55	0.0805
1.5000	−0.1643	16.85	0.0776	−0.3770	16.34	0.0806
1.7500	−0.2797	16.65	0.0776	−0.4351	16.17	0.0809
2.0000	−0.3933	16.47	0.0776	−0.5040	16.05	0.0813
3.0000	−0.7878	15.90	0.0792	−0.8663	15.89	0.0843
4.0000	−1.1531	15.60	0.0826	−1.2123	15.75	0.0893
5.0000	−1.4839	15.64	0.0877	−1.4526	15.62	0.0960
6.0000	−1.7273	15.67	0.0942	−1.6097	15.67	0.1043
7.0000	−1.8811	15.88	0.1015	−1.6905	15.90	0.1133
8.0000	−1.9500	16.20	0.1089	−1.6968	16.21	0.1221
9.0000	−1.9418	16.52	0.1157	−1.6492	16.61	0.1297
10.0000	−1.8837	16.86	0.1216	−1.5695	17.09	0.1357
11.0000	−1.8026	17.27	0.1265	−1.4788	17.62	0.1395
12.0000	−1.7129	17.75	0.1303	−1.4011	18.21	0.1408
13.0000	−1.6228	18.31	0.1328	−1.3516	18.83	0.1402
14.0000	−1.5405	18.94	0.1343	−1.3280	19.47	0.1383
15.0000	−1.4692	19.59	0.1350	−1.3249	20.06	0.1359
16.0000	−1.4075	20.21	0.1349	−1.3348	20.58	0.1333
17.0000	−1.3522	20.78	0.1345	−1.3518	21.01	0.1308
18.0000	−1.2999	21.26	0.1338	−1.3720	21.36	0.1286
19.0000	−1.2490	21.68	0.1330	−1.3935	21.63	0.1268
20.0000	−1.2007	22.07	0.1321	−1.4151	21.85	0.1253
21.0000	−1.1559	22.44	0.1313	−1.4361	22.05	0.1240

y = age in years.

*0.0767 = 4 weeks.

L = Box-Cox power transformation required to remove the skewness of the distribution.

M = median.

S = coefficient of variation.

**Figure 1:**
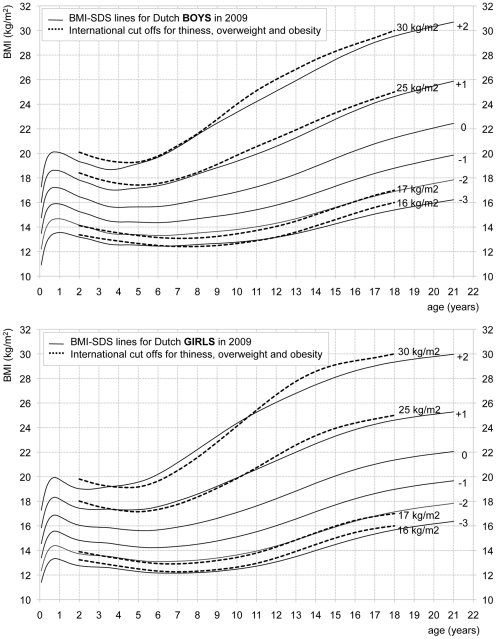
BMI distribution for Dutch boys and girls in 2009. The solid lines represent −3 (0.1^th^), −2 (2.3^th^), −1 (16^th^), 0 (50^th^), +1 (84^th^), +2 (97.7^th^) SDS. The dotted lines represent international cut-off values for obesity (30 kg/m2), overweight (25 kg/m2), thinness grade II (17 kg/m2) and thinness grade III (16 kg/m2) [16], [29]. Effective degrees of freedom (edf) of the model of boys: 12 (M curve), 3 (S curve), 3 (L curve). Effective degrees of freedom (edf) of the model of girls: 9 (M curve), 4 (S curve), and 3 (L curve).

Figure 1 shows the BMI distribution that correspond to the fitted LMS values for both sexes, including the 0, ±1, ±2, and −3 SDS lines. For comparison, the charts include the international cut-off values for thinness grade II and III, overweight and obesity [16], [29]. The distribution was highly skewed. From the age of three years on, the distance between the +1 and +2 SDS lines was 1.5 to 3 times as wide as between the −1 and −2 SDS lines at all ages. The +2, +1, −2 and −3 SDS lines corresponded well to the international cut-off values for respectively obesity, overweight, thinness grade II and III, although the IOTF cut-off values for obesity were a bit higher than the +2 SDS line for girls from the age of 11 years onward. In general, the median (0 SDS) curves for boys and girls were very similar, although BMI values for boys up to one year of age were slightly higher and during puberty slightly lower than for girls.

Table 2 presents the mean BMI for boys and girls at all ages in 1980, 1997 and 2009. The mean BMI rose in both boys and girls. Since 1997, mean BMI in boys increased with 0 to 3%, and compared with 1980, it was up to 5% higher. In girls, mean BMI rose 0 to 2.4% since 1997 and was up to 4.5% higher than in 1980. The rise in mean BMI was seen in boys aged four years and older and in girlsz from the age of three years onwards, with the largest relative increase occurring around the age of eight to ten years.

**Table 2:** Median (P50) BMI in 1980, 1997 and 2009 by age and sex.

	boys			girls		
age (y)	1980	1997	2009	1980	1997	2009
2.0	16.6	16.4	16.5	16.2	16.1	16.1
3.0	15.9	15.9	15.9	15.7	15.7	15.9
4.0	15.5	15.6	15.6	15.4	15.5	15.8
5.0	15.3	15.5	15.6	15.2	15.4	15.6
6.0	15.2	15.5	15.7	15.1	15.5	15.7
7.0	15.3	15.6	15.9	15.3	15.7	15.9
8.0	15.5	15.8	16.2	15.5	16.0	16.2
9.0	15.7	16.1	16.5	15.9	16.3	16.6
10.0	16.1	16.4	16.9	16.4	16.7	17.1
11.0	16.6	16.8	17.3	16.9	17.2	17.6
12.0	17.1	17.3	17.8	17.5	17.8	18.2
13.0	17.7	17.9	18.3	18.1	18.5	18.8
14.0	18.3	18.5	18.9	18.7	19.2	19.5
15.0	18.8	19.2	19.6	19.3	19.8	20.1
16.0	19.4	19.9	20.2	19.8	20.3	20.6
17.0	20.0	20.4	20.8	20.3	20.8	21.0
18.0	20.5	20.9	21.3	20.7	21.2	21.4
19.0	21.0	21.4	21.7	21.1	21.5	21.6
20.0	21.5	21.8	22.1	21.6	21.8	21.9
21.0	NA	22.1	22.4	NA	22.1	22.1

y = age in years.

NA = not available.

**Figure 2:**
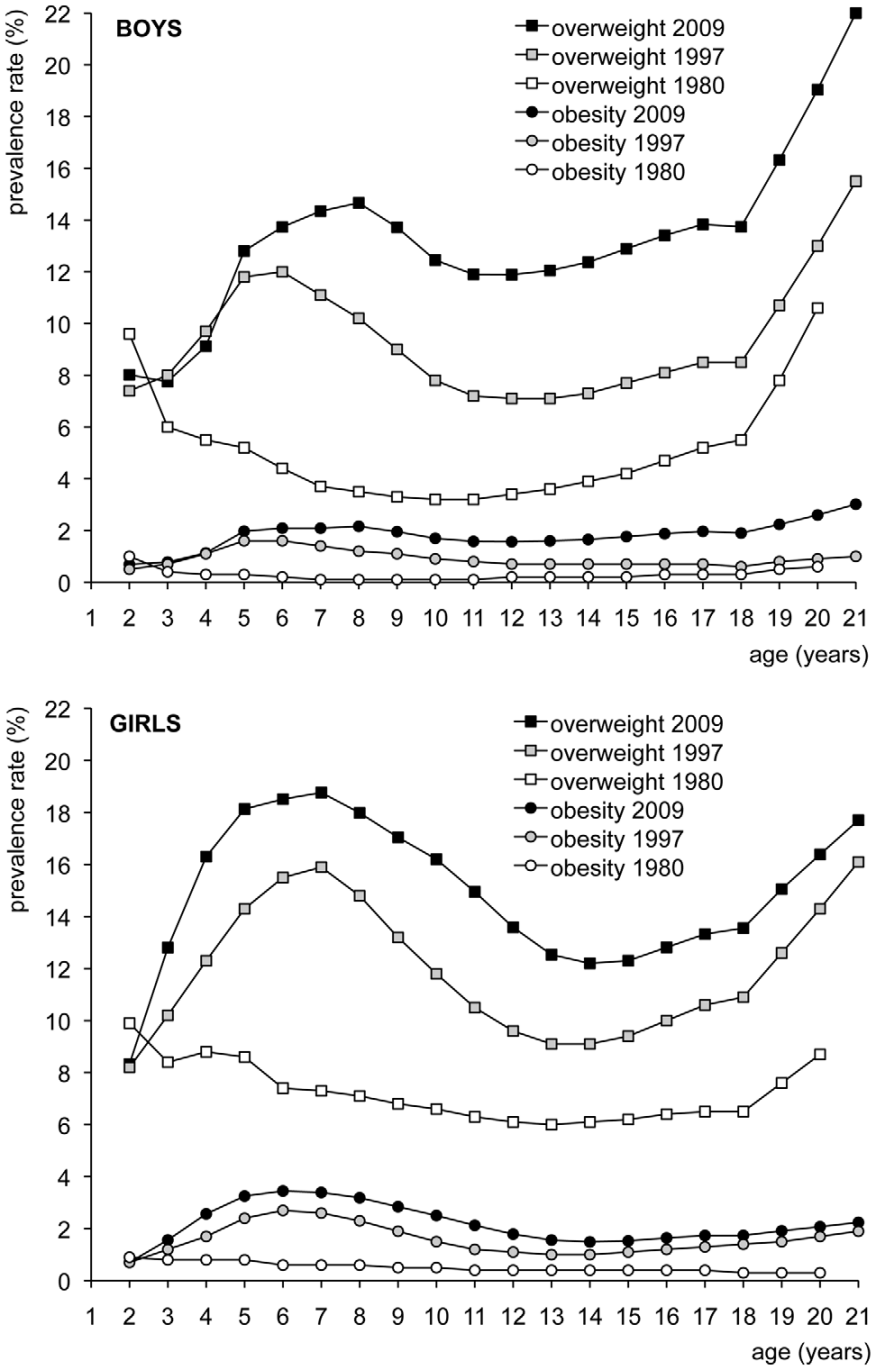
Prevalence of overweight and obesity in Dutch boys and girls according to international cut-off values [**16**].

Table 3 compares overweight and obesity prevalence rates in 2009 with 1980 and 1997 for Dutch boys and girls [3], [4]. From this table we calculated an increase of 20–40% in overweight and of 40–100% in obesity since 1997. Figure 2 graphs these prevalence rates by age. At the age of two years, overweight and obesity prevalence rates in 2009 were slightly lower than in 1980 and similar to those in 1997. However, after the age of two years, the 2009 prevalence rates exceeded the rates observed in 1980 and 1997. For girls, the gap started to widen from the age of two years, whereas for boys this occurred from the age of four years. The largest gap with the previous studies was found around the ages of seven and eight years, when 15–19% of the children were overweight and 2–3% were obese. At some ages, we observed a three to four fold increase in the amount of overweight since 1980. For obesity, this ratio was even higher.

**Table 3:** Prevalence rates (%) of overweight (including obesity) and obesity according to IOTF cut-off values [16] in 1980, 1997 and 2009 by age and sex.

	overweight						obesity					
	boys			girls			boys			girls		
age (y)	1980	1997	2009	1980	1997	2009	1980	1997	2009	1980	1997	2009
2.0	9.6	7.4	8.0	9.9	8.2	8.3	1.0	0.5	0.7	0.9	0.7	0.7
3.0	6.0	8.0	7.8	8.4	10.2	12.8	0.4	0.7	0.8	0.8	1.2	1.6
4.0	5.5	9.7	9.1	8.8	12.3	16.3	0.3	1.1	1.1	0.8	1.7	2.6
5.0	5.2	11.8	12.8	8.6	14.3	18.1	0.3	1.6	2.0	0.8	2.4	3.3
6.0	4.4	12.0	13.7	7.4	15.5	18.5	0.2	1.6	2.1	0.6	2.7	3.4
7.0	3.7	11.1	14.3	7.3	15.9	18.8	0.1	1.4	2.1	0.6	2.6	3.4
8.0	3.5	10.2	14.7	7.1	14.8	18.0	0.1	1.2	2.2	0.6	2.3	3.2
9.0	3.3	9.0	13.7	6.8	13.2	17.0	0.1	1.1	2.0	0.5	1.9	2.8
10.0	3.2	7.8	12.5	6.6	11.8	16.2	0.1	0.9	1.7	0.5	1.5	2.5
11.0	3.2	7.2	11.9	6.3	10.5	15.0	0.1	0.8	1.6	0.4	1.2	2.1
12.0	3.4	7.1	11.9	6.1	9.6	13.6	0.2	0.7	1.6	0.4	1.1	1.8
13.0	3.6	7.1	12.0	6.0	9.1	12.5	0.2	0.7	1.6	0.4	1.0	1.6
14.0	3.9	7.3	12.4	6.1	9.1	12.2	0.2	0.7	1.7	0.4	1.0	1.5
15.0	4.2	7.7	12.9	6.2	9.4	12.3	0.2	0.7	1.8	0.4	1.1	1.5
16.0	4.7	8.1	13.4	6.4	10.0	12.8	0.3	0.7	1.9	0.4	1.2	1.6
17.0	5.2	8.5	13.8	6.5	10.6	13.3	0.3	0.7	2.0	0.4	1.3	1.7
18.0	5.5	8.5	13.7	6.5	10.9	13.6	0.3	0.6	1.9	0.3	1.4	1.7
19.0	7.8	10.7	16.3	7.6	12.6	15.1	0.5	0.8	2.2	0.3	1.5	1.9
20.0	10.6	13.0	19.0	8.7	14.3	16.4	0.6	0.9	2.6	0.3	1.7	2.1
21.0	NA	15.5	22.0	NA	16.1	17.7	NA	1.0	3.0	NA	1.9	2.2
2.0–21.0*	5.1**	9.4	13.3	7.2**	11.9	14.9	0.3**	0.9	1.8	0.5**	1.6	2.2

y = age in years.

NA = not available.

* = mean prevalence for children aged 2–21 years.

** = mean prevalence for children aged 2–20 years, as data for 21 years olds in 1980 were not available.

From Table 3 we calculated an increase of the relative proportion of obesity among overweight boys from 6% in 1980, through 10% in 1997, up to 14% in 2009. In girls, similar figures were found, starting at 7% in 1980, rising to 14% in 1997 and reaching 15% in 2009. Although the rise from 1997 to 2010 was not statistically significant (p = 0.08 for boys and p = 0.491 for girls), there was a rising trend in the severity of overweight.

**Figure 3:**
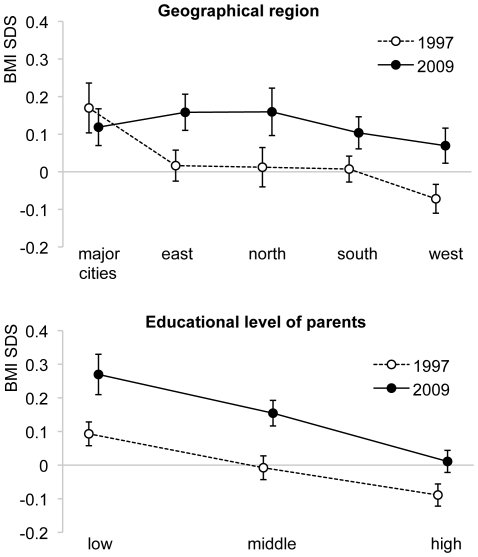
Mean BMI SDS per geographical regions and parental education in 1997 and 2009. Mean BMI SDS with 95% confidence interval per geographical region and level of parental education in 1997 and 2009.

Figure 3 presents the effect of geographical region and educational level of the parents on BMI SDS and compares these figures in 2009 with 1997. The mean BMI SDS of children whose parents had a low education was clearly higher than in those of higher educated parents. BMI SDS increased in all groups compared to 1997, but the increase was smallest in children of parents with a high educational level. Across the geographical regions we saw no large differences in BMI SDS in 2009. In 1997, in contrast, the BMI SDS in the major cities was much higher and in the west of the country it was lower than in the other regions. When we looked within the regions, we noticed a significant increase in BMI SDS since 1997 in all regions except for the major cities. In the major cities, the mean BMI SDS in 2009 even lay beneath 1997, although their 95% confidence intervals overlap. This corresponded well with the overweight prevalences that stabilized in the major cities (14.1% in 1997 vs. 14.4% in 2009, p = 0.796), but rose substantially in the other regions (9.0% in 1997 vs. 12.9% in 2009, p<0.001).

## Discussion

The percentage of Dutch children with overweight and obesity in 2009 is higher than in 1997. Currently, 13–15% of the Dutch children are overweight. This is a two to three fold increase of the 1980 overweight prevalence rates. Two percent of the Dutch children are obese, which is four to six times the prevalence found in 1980. Further, compared to 1997, when the Dutch overweight epidemic became apparent, a large increase in both overweight and obesity has occurred. Although the Dutch prevalences are still relatively low compared to other developed countries [30], [31], our data strongly indicate that overall overweight and obesity in the Netherlands are still increasing, not only in the number of children classified as overweight or obese, but also in the relative proportion of obesity among those children who are overweight.

Another important finding in this study is that the prevalence of overweight among Dutch children stabilized in the major cities. This finding is supported by a recent study in The Hague, one of these major cities. No increasing trend was observed in the prevalence of overweight in Dutch boys in The Hague between 1999 and 2007 and a decrease in prevalence was found among Dutch girls [32]. Many present day prevention strategies in the Netherlands aim at populations in the major cities and this stabilization may be the effect of these programs. We wish to add that our data do not allow any conclusions about these programmes' effectiveness. In addition, it should be noted that the overweight prevalence in 1997 was much higher in the major cities than in the rest of the country and that the stabilization caused the major cities to blend in with the other regions regarding overweight prevalence. Nevertheless, these may be the first signs that the overweight prevalence begins to level off among those at highest risk.

A study by Van den Hurk [33] predates the present study by about five years. Van den Hurk et al. was based on a convenience sample and did not feature quality assurance like ours with respect to the measurement methodology. Nonetheless, they found similar overweight prevalences in the Netherlands around 2003 as we now find in 2009. This could mean that the overweight prevalence has stabilized after 2003, and that the major increases in prevalence must have taken place before 2003. This is in line with the evidence that has emerged from several developed countries suggesting that the rise in the prevalence has slowed appreciably, or even plateaued between 1995 and 2008 [34]. Although most of their overweight and obesity prevalences are much higher than in our study, these may be small glimpses of hope that the worst in terms of continuing increase might be over.

Although various factors have been identified as possible causes for the increase in childhood overweight and obesity, the exact cause of the current rise is unknown, and could also differ between people. It is, however, known that overweight is a result of an imbalance between energy intake and expenditure, and it is generally assumed that the overweight epidemic is mainly due to environmental factors, as genetic changes would not occur at this rate [35]. Parental knowledge of causes of overweight and of healthy food is often insufficient. A Dutch study showed that only 34% of the parents of young children had sufficient knowledge on causes of overweight, 61% on the consequences of overweight, and 49% on healthy food [36]. Other studies in the Netherlands found that 50% of the parents did not recognize overweight in their child and 87% of the parents with an overweight child did not worry about their child's weight [37], [38]. To tackle the overweight problem, intervention programs that lead to long-lasting lifestyle changes and reductions in the prevalence of overweight and obesity are needed. So far, intervention programs have had little success. Although it is not a randomized trial, the French total-community approach EPODE seems promising. First results from two small towns showed that between 2002 and 2004 the prevalence of overweight decreased from 13.2% to 8.8%, and was significantly lower than in nearby comparison towns [39]. Future results from EPODE, which has been extended to more than 200 towns in Europe, and other total-community programs should provide more information about the effectiveness of an environmental approach that involves and activates entire neighbourhoods and communities.

A recent longitudinal study provided evidence of the importance of the age period two to six years for the risk of becoming overweight as an adult [11]. It is remarkable that in our study at the age of two years, the overweight prevalence has not changed over the last 30 years. It could mean that prevention efforts aimed at children before the age of two years are in fact too early in life. Moreover, our data show peak prevalences at the ages of seven and eight years, with the steepest increase before the age of five. This pattern suggests that life style changes may have occurred between 1980 and 2009 that especially affect children between 2 and 6 years of age. Our findings are thus entirely consistent with, and in fact reinforce, the findings by De Kroon, and emphasize the importance of early prevention and intervention regarding childhood overweight and obesity.

For monitoring BMI in children in the Netherlands, up until now reference chart describing the BMI distribution in the population, with or without IOTF cut-off values, have been used [40]. As the BMI continued to rise, the population distribution becomes less suitable for monitoring purposes. We therefore decided to create normative BMI reference charts that only include international cut-off values for overweight, obesity, thinness grade II and thinness grade III, which correspond to a BMI of 30, 25, 17 and 16 kg/m2 respectively at the age of 18 years [16], [29], [41].

### Conclusion

The results of the Fourth Dutch Growth Study in 1997 created awareness regarding the problem of overweight and obesity in the Netherlands. Many preventive programs have been implemented and are currently being evaluated. Our study shows that despite all the efforts, overall overweight and obesity prevalences have increased since 1997. However, the finding that the prevalence in the major cities, where many of these preventive programs have been implemented on a large scale, has stabilized, may imply that increased awareness, prevention and intervention strategies have started to turn the rising trend in overweight around. Continuation of these efforts is needed to fight the obesity epidemic nationwide, as in absolute levels there is still a considerable societal and health problem. Monitoring of the development of childhood overweight and obesity on the national level remains important to track developments in childhood overweight and obesity over the next decades.
